# Visualization-Driven Time-Series Extraction from Wearable Systems Can Facilitate Differentiation of Passive ADL Characteristics among Stroke and Healthy Older Adults

**DOI:** 10.3390/s22020598

**Published:** 2022-01-13

**Authors:** Joby John, Rahul Soangra

**Affiliations:** 1 Schmid College of Science and Technology, Chapman University, Orange, CA 92866, USA; jjohn@chapman.edu; 2 Fowler School of Engineering, Chapman University, Orange, CA 92866, USA; 3 Crean College of Health and Behavioral Sciences, Chapman University, Irvine, CA 92618, USA

**Keywords:** recurrent neural network (RNN), activities of daily living (ADL), long short-term memory (LSTM), time-series extraction, stroke, body mass index (BMI)

## Abstract

Wearable technologies allow the measurement of unhindered activities of daily living (ADL) among patients who had a stroke in their natural settings. However, methods to extract meaningful information from large multi-day datasets are limited. This study investigated new visualization-driven time-series extraction methods for distinguishing activities from stroke and healthy adults. Fourteen stroke and fourteen healthy adults wore a wearable sensor at the L5/S1 position for three consecutive days and collected accelerometer data passively in the participant’s naturalistic environment. Data from visualization facilitated selecting information-rich time series, which resulted in classification accuracy of 97.3% using recurrent neural networks (RNNs). Individuals with stroke showed a negative correlation between their body mass index (BMI) and higher-acceleration fraction produced during ADL. We also found individuals with stroke made lower activity amplitudes than healthy counterparts in all three activity bands (low, medium, and high). Our findings show that visualization-driven time series can accurately classify movements among stroke and healthy groups using a deep recurrent neural network. This novel visualization-based time-series extraction from naturalistic data provides a physical basis for analyzing passive ADL monitoring data from real-world environments. This time-series extraction method using unit sphere projections of acceleration can be used by a slew of analysis algorithms to remotely track progress among stroke survivors in their rehabilitation program and their ADL abilities.

## 1. Introduction

In the United States, stroke is exceptionally prevalent; approximately 3% of the adult population has experienced one [1]. Stroke has led to estimated direct and indirect costs of USD 68.9 billion in 2009, and projections suggest an upward trend as the population ages [1]. Stroke is also one of the leading causes of severe and long-term disability in adults and is associated with limb weakness and paralysis. Individuals with stroke are often dependent on caregivers for assistance with activities of daily living (ADL) [2,3], and their performance during ADL is considered a clinical measure of disability [4,5,6,7]. The ADL loss among individuals with stroke is found even before the onset of stroke [8]. Thus, ADL performance is the primary functional biomarker in stroke rehabilitation assessments because of its objectivity, simplicity, and relevance to this patient population [9]. Currently, disability assessment among stroke survivors involves subjective scoring such as the Frenchay activities index (FAI) [10] and Gutmann scaled ADL assessment [11,12]. ADL monitoring is necessary among individuals with stroke [13,14] since it is associated with after-stroke depression (ASD) [15,16] and decreased quality of life (QOL) [17]. Wearable systems allow multi-days of continuous tracking of movement data and can comprehensively depict the dynamic health status of patients. Wearable sensors are becoming a ubiquitous tool to enhance the QOL and assess successful rehabilitation among stroke survivors [18]. Although wearable devices are available in various miniature forms, advanced algorithms to analyze a massive continuum of datasets are lacking. Sensor-based multi-day human activity monitoring generates large datasets [19] from natural settings. For example, at a data sampling rate of 100 Hz, one accelerometer sensor can provide over 8.6 million samples over a single day. Recently, Borton and coworkers reported that activity energy expenditure derived from wrist-worn wearables can serve as a good predictor of clinical average levels of physical activity in older adults (*n* = 2472) and thus objectively measure physical activity in a population-based study [20]. Although these vast datasets have rich mobility information, signal processing algorithms to analyze such enormous datasets for extracting essential features relevant to one’s mobility are lacking. Today, wearable sensors have limited use due to the lack of intuitive algorithms that can reliably organize and interpret movement acceleration data during daily life activities. This inference of acceleration data is also essential to enable proactive health management and to develop formal ontologies between research groups [21]. Recently, Chen and coworkers reported classification of ADL activities using wearable sensors and machine learning in individuals with stroke with high accuracy [22]. We have earlier reported that frequency and transition-related information from longitudinal multi-day datasets can differentiate obese versus non-obese [23] and older versus younger adults [24]. In this study, we developed a novel visualization-based time-series extraction and analysis algorithm that can capture the entirety of the data and delineate critical information from the long-motion datasets. Visualization of long movement datasets through visual characteristics can quickly reveal activity zones related to sleeping, walking, and other activities. This study contributes to the extraction of visualization-driven time series from passive longitudinal multidimensional sensor data and applies machine learning algorithms to differentiate stroke-related movement asymmetry. We investigated recurrent neural networks such as the long short-term memory (LSTM) model to determine its performance and stroke versus healthy movement discrepancies.

This study essentially explored new visualization-based methods to select time series and deployed deep learning algorithms to distinguish stroke and healthy counterparts from multi-day longitudinal datasets. This study opens new avenues in differentiating stroke ADLs from healthy peers by collecting sensor data unobtrusively from natural home settings. Additionally, the visualization method to extract time series is innovative and has broader impacts on information extraction from passive wearable sensors. This is critical since ML classifiers can track the effects of clinical rehabilitation on stroke ADL performance.

## 2. Materials and Methods

### 2.1. Data Visualization Method for Time-Series Extraction

**Figure 1:**
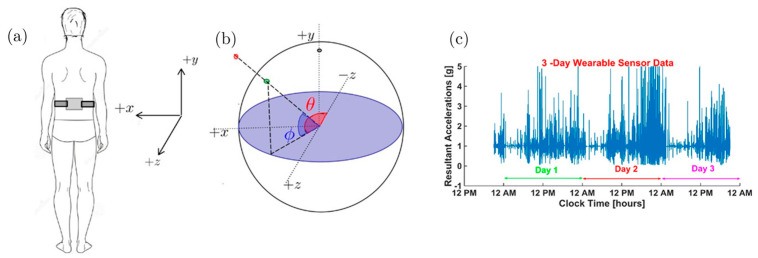
(**a**) Schematic diagram of an IMU worn by a participant. In this configuration, the positive acceleration in the *x*-direction (+a_x_) is directed toward the left side, the +a_y_ direction is pointed vertically upwards (opposing gravity), and the +a_z_-direction is pointed in the posterior direction. (**b**) Unit acceleration sphere (UAS): The axes refer to the acceleration axis in a sensor-centric left-handed coordinate system. The equatorial plane is shown in blue color. A sample acceleration point [*a_x_*, *a_y_*, *a_z_*] is shown as a red dot at some time. Its projection on the UAS is represented as the green dot. (**c**) Longitudinal 3-day resultant acceleration data.

Three days of inertial sensor data points from accelerometers contain 25.92 × 10^6^ samples for each channel (tri-axial accelerometers consisting of *x*, *y*, and *z* axes) and timestamps. The initial task is to visualize the entirety of the acceleration data to aid further analysis. However, it is challenging to map complete data samples in their entirety to provide information for activities performed and their magnitudes for comparison among stroke and healthy groups. Since the resultant acceleration of 3-dimensional accelerations is independent of the sensor orientation (how participant-oriented sensors in the home environment may differ), the resultant acceleration vector offered a basis for robust analysis. For example, when the participant is stationary (with no or minimal movement), regardless of the orientation of the sensor, the resultant acceleration is unity. Resultant accelerations can be projected on a unit acceleration sphere (UAS) to comprehensively visualize all movement artifacts in a single shot. All ADLs such as walking, jogging, reaching, climbing stairs, sitting, standing, laying down, etc., can be viewed as movements happening in the vicinity of a stationary stance position in different vector directions. An IMU worn at the torso (Figure 1a) could provide rich information about a person’s movement characteristics since the torso carries 2/3rds of body weight and represents the whole-body center of mass (COM). To visually represent the resultant accelerometer data from different ADL, the Cartesian coordinates ax, ay, and az of the accelerometer were transformed to spherical coordinates (ρ,ϕ,θ) where ϕ ∈ [−*π*, *π*] is the azimuthal angle, *θ* ∈ [−*π*/2, *π*/2] is the polar angle measured from the equator, and *r* is the radial distance from the origin. In Figure 1b, a sample acceleration vector is shown projected onto the UAS. In Figure 1c, the timeseries of the magnitude of the acceleration vector over 3 days is shown. Such a visualization of the data is not very helpful in understanding movement characteristic of stroke and healthy individuals. 

#### 2.1.1. Unit Acceleration Sphere (UAS)

We propose the visualization of this large passively collected dataset arising from three consecutive days of activities by projecting the acceleration data onto the sphere of unit radius in the acceleration space, UAS. The axes X, Y, and Z (Figure 1b) refer to the sensitive axes of the accelerometer, and *a_x_*, *a_y_*, and *a_z_* represent the components of acceleration along each of these directions. The equatorial plane is shown in shaded blue in Figure 1b. A sample point is represented as a red dot, and its projection on the unit sphere is described as a green dot. The resultant acceleration is defined in Equation (1).

The acceleration vector **a** = [*a_x_*, *a_y_*, *a_z_*] represented in Cartesian coordinates is transformed to a vector in spherical coordinates [ρ,ϕ,θ], where ϕ∈[−π,π] is the azimuthal angle, θ ∈ [−π/2,π/2]  is the polar or elevation angle measured from the equator, and *ρ* is the radial distance from the origin. The projection of every sample point onto the unit sphere is obtained by setting the radial coordinate to one (ρ = 1) while keeping the azimuthal (ϕ) (Equation (2)) and polar angle (θ) (Equation (3)) of the original signal point. This 3-dimensional unit sphere shows an equatorial *x*–*z* plane and the 3 axes since 3 complete days of resultant acceleration do not reveal much movement information (Figure 1c). A sample of the ADL dataset can be projected as a red point and its intersection as a green point on the unit sphere (Figure 1b)


(1) $$a=\left|a\right|=\sqrt{\left(a_{x}^{2}+a_{y}^{2}+a_{z}^{2}\right)}$$

(2) $$\theta =\tan^{-1}\left(\frac{a_{y}}{\sqrt{a_{x}^{2}+a_{z}^{2}}}\right)$$

(3) $$\phi =\tan^{-1}\left(\frac{a_{x}}{-a_{z}}\right)$$

Every data sample point is projected onto the UAS by setting the radial coordinate *ρ* = 1 and leaving the original sample point’s azimuthal (ϕ) and polar angle (θ) of the original sample point unchanged. Thus, we are able to parametrize the UAS with the two variables ϕ and θ to create two new features for each dataset that help us in visualizing the entire dataset in a manner that helps us gain some insights into the data, as described below.

#### 2.1.2. Task-Agnostic Time-Series Extraction for Recurrent Neural Networks (RNNs)

**Figure 2:**
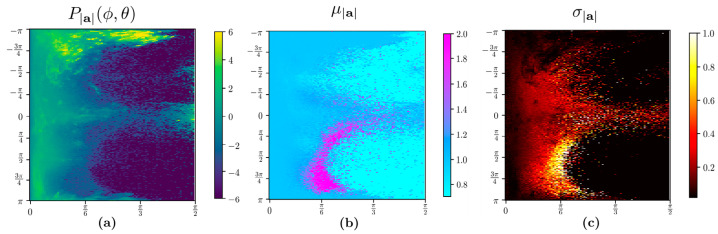
We can extract interesting statistics such as the probability distribution (**a**), means (**b**), and standard deviation (**c**) of the acceleration vector across the UAS by discretizing the Φ and θ features defined in Equations (2) and (3) (shown here using three different color maps). This representative data corresponds to the southern hemisphere of the UAS of an individual with stroke. The different colormaps represent different statistics.

**Figure 3:**
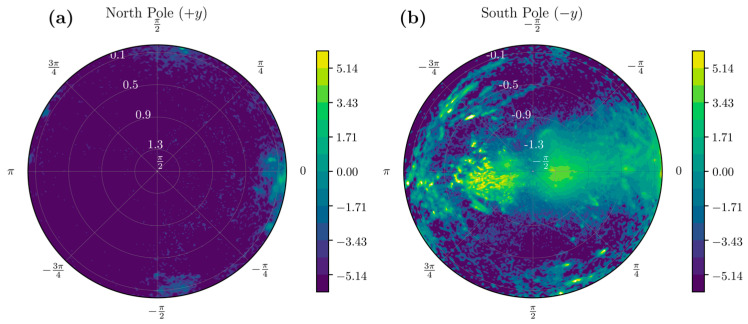
A visual representation of acceleration data from 3 days of continuous sensor wearing (25.92 million data samples) for a healthy participant projected onto the (**a**) upper and (**b**) lower hemispheres of the unit *g* acceleration sphere (UAS) when viewed along the y-axis. The color bars represent the log of the probability density of the projection of the acceleration onto the UAS.

One of the significant challenges is extracting information-rich data from extensive day-long time-series data to analyze human movement characteristics. For example, the data collected during night-time sleep may not reveal stroke’s gait and movement-related asymmetry characteristics. In essence, the acceleration profiles during the sleep or stationary phase are similar between post-stroke survivors and healthy counterparts. However, the two groups expected a marked difference in acceleration patterns during active daytime movements. Elevation and azimuthal angle features derived from the visualization-based time-series extraction method on UAS can help extract meaningful information-rich time-series data, as described below (Figure 2). This extracted time series can be an input to train recurrent neural networks (RNNs) to classify pathological stroke-related movement datasets. However, there are multiple approaches to extracting valuable data in longitudinal healthcare datasets [24]. In this study, we propose to utilize visualization techniques to identify essential activity data chunks represented on UAS (Figure 3). The two-dimensional surface of the UAS is discretized in the ϕ ∈ [−π,π] and θ ∈ [−π/2,π/2] variables into 360 and 180 bins, respectively, such that each bin corresponds to 1° in both variables. Every sample projected on the UAS is assigned a bin number [i, j] to which it belongs where i, j are integers i ∈ [1, 360] and j ∈ [1, 180]. Once bin numbers are assigned, we can compute the empirical probability distribution of the acceleration magnitude |**a**| across the bins, the distribution of mean |**a**|, and the distribution of the standard deviation of |**a**|. In the raw form, these distributions look like 2D matrices (Figure 2).

Here, even though three different statistics are extracted from the same dataset, the 2D matrix form does not help in relating the data to the physical orientation of the acceleration vectors. We look at the same data on a polar plot (Figure 3) such that it corresponds to viewing the UAS from the north and south poles. Most of the data is around the south pole of the UAS, and this corresponds to acceleration vector orientation during vertical rest state. The yellow shades in this figure indicate orientations that are visited more frequently by the participant.

**Figure 4:**
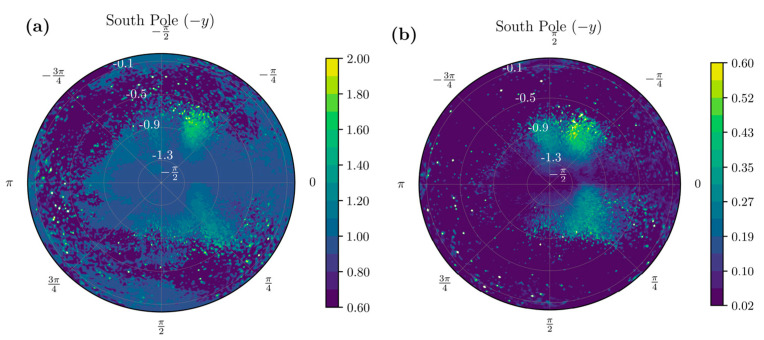
Visual representation of mean and standard deviation of resultant accelerations magnitude across various orientations when performing ADLs. (**a**) Distribution of means of acceleration samples for every orientation across the southern hemisphere of the UAS. Higher means indicate high-acceleration movements in that orientation. (**b**) The variability of the motion for each orientation is shown. The color bars represent the log of the probability density of the projection of the acceleration onto the UAS.

In addition to the probability distribution, the distribution of the mean magnitude of acceleration for each orientation (ϕ, θ) across the southern hemisphere of UAS (Figure 4a) helps us identify zones of high amplitude movements. Similarly, the variability of acceleration magnitude for each orientation (Figure 4b) could provide insights into zones of high variability and repeated movements. We can use these insights to develop criteria to filter the full dataset into samples that contain the most information about movement characteristics.

(1) Considering that the resultant acceleration vector **a** = [0, −1, 0] *g*’ in the stationary standing position corresponds to the south pole on the UAS, we look for data only in the southern hemisphere, or θ < 0.
(2) Considering movements when in standing position, we look for data such that θ < −π/3, a neighborhood of the south pole, i.e. we consider samples with  θ∈[−π/2,−π/3].
(3) The algorithm selected all bins with a standard deviation of the bin σ**_|_**_a**|**_ ≥ 0.02 and the mean |µ**_|a|_** − g| > 0.02 for input to recurrent neural networks. The rationale is that the bin corresponding to orientations where resultant acceleration signals show near-zero standard deviations and mean close to gravitational acceleration UAS (|**a**| = 1g) fits the rest of the scenarios and, therefore, there is not much movement-related information. For example, these scenarios can be regular breathing artifacts and posture re-orientations during sitting, standing, or other static postures. Furthermore, only daytime samples collected between 7 a.m. and 8 p.m. were considered since subjects were more active when awake during the day. The data samples that met the above time constraints were split into continuous 3 s time-series samples using the timestamp. Since postural transitions typically take up to 3 s, choosing this window size can capture such transitions. We used such 3 s long time-series samples with thirteen features to train a deep long short-term memory (LSTM) neural network using twelve features include: tri-axial acceleration, tri-axial angular velocity, resultant acceleration, acceleration components on the UAS, polar angle θ, and azimuthal angle ϕ. We obtained 37,383 and 19,067 data samples from healthy and stroke participants, respectively. Each sample has 3 s worth of data containing 11.21 × 10^6^ samples for the healthy group and 5.72 × 10^6^ samples for individuals with stroke. With such large numbers, the Z-test comparison yields extremely small *p*-values, and therefore, we report the effect size using Cohen’s d. The mean and standard deviations of these features and the effect size measured by Cohen’s d are listed in Table 1 below. We note that most of the features have only a small difference in the two populations, as evidenced by the small Cohen’s d values.

We randomly shuffled these data samples and 80% of the data samples to train the network and the remaining 20% to validate the network. We did not test the model separately for performance evaluation on stroke and healthy groups. This work aims to demonstrate the efficacy of visual techniques in filtering data samples for machine learning and classification. Table 2 below shows the input and dense hidden layers and out layer as implemented in TensorFlow.

#### 2.1.3. Higher Acceleration Fraction

In order to compare the number of high-acceleration movements in the two groups, we consider the ratio of the number of samples with magnitude |**a**| greater than −2g to the total number of samples obtained after filtering the data by the above-mentioned constraints. Since such movements with |**a**| > −2g are expected to be low compared to the samples collected throughout the day, we consider the logarithm of the ratio. We define the higher-acceleration fraction as:

(4) $$Higher\;Acceleration\;Fraction=\log\left(\frac{Samples>2g}{Total\;samples}\right)$$

We expect this measure of high-acceleration movements to be considerably lower in people with stroke compared to their healthy counterparts, owing to the limitations imposed by their pathophysiology.

#### 2.1.4. Stroke-Related Asymmetry Quantification

Individuals with stroke have movement asymmetry due to neural pathology [25]. This asymmetry may reflect movement signals’ high-acceleration fraction (|**a**|> 2*g*). Affected versus unaffected side movement can be distinguished by the azimuthal angle ϕ. The number ($n_{R}$) of high-acceleration movements on the right side (ϕ<0) and the number of samples on ($n_{L}$) left side (ϕ≥0) are substantially different in asymmetric movements. Since healthy individuals are more symmetric, we expect this $n_{L}/n_{R}$ ratio will be closer to unity. This asymmetry ratio is farther from unity among individuals with stroke. The affected limb may produce lower accelerations and induce rigidity in movements. One side may have more data samples than the other. Additionally, these ratios could be orders of magnitude different in stroke and healthy subjects. To compare these numbers in the two populations, we consider the logarithm of the ratio. We define the acceleration asymmetry index (AAI) in Equation (5) below.

(5) $$Acceleration\;Asymmetry\;Index\;\alpha =\log\left(\frac{n_{R}}{n_{L}}\right)$$

Thus, we expect AAI to be close to zero for healthy subjects and farther from 0 for stroke subjects. 

Sedentary Behavior: A decline in high-acceleration movements is indicative of sedentary behavior. Log of the fraction of samples: $\log\left(n_{a}/N\right)$, where $n_{a}$ is the number of data samples with |**a**| > 2 *g*, and N is the total number of data samples at daytime and the elevation criteria (between 7 a.m. and 8 p.m., θ < −π/3).

#### 2.1.5. Movement Transitions and Activities in Frequency Bands

Three days of human movement accelerometric data can quantify movement transitions and frequency of movement signal [21]. Earlier reported algorithms identified sleep, movement transitions, and frequency during daytime activities [21].

Sleep Data Identification: We evaluated resultant acceleration in the XZ plane, as shown in Equation (4) below [23], to identify sleep signals.

(6) $$R_{a,xz}=\sqrt{a_{x}^{2}+a_{z}^{2}}$$

We utilized a 1 s moving window to identify sleep data, with a threshold of mean ± variance of *R*_*a,xz*_ from time series bounded between 0.97*g* and 1.02*g* [23] acceleration ranges. 

Movement Transitions: Movement transitions were defined using the information of peaks and duration between peaks [23]. We utilized the resultant acceleration to evaluate the (i) number of transitions, (ii) sleep hours, (iii) maximum, (iv) minimum, (v) root mean square (rms), (vi) range, and (vii) duration of acceleration within a transition [23]. We generated wavelet-based frequency analysis algorithms using a complex Morlet wavelet (CMW) by multiplying a complex sine wave with a Gaussian wave.

Activity Amplitude (AA): The activity amplitudes were defined similarly to our previous work [23]. We computed detrended resultant acceleration (DRA) signals by subtracting gravitational accelerations from resultant accelerations and computed its absolute value. Then, activity categorizing thresholds were defined similarly to thresholds defined earlier [23,24]. The activity amplitude is the time integral of DRA signals over the period [23]. Activity amplitudes were categorized amplitude wise (low, medium, high) and in time zones as Time Zone 1 (12 a.m.–5:59 a.m.), Time Zone 2 (6 a.m.–11:59 a.m.), Time Zone 3 (12 p.m.–5:59 p.m.), and Time Zone 4 (6 p.m.–11:59 p.m.) [23].

### 2.2. Experiment and Data Collection

The study recruited fourteen healthy and fourteen individuals with stroke. All participants signed the written informed consent approved by the Institutional Review Board at Chapman University. Table 3 shows the participants’ anthropometric information. Table 4 provides the Fugl–Meyer scores of the participants. The participants wore a Dynaport sensor (McRoberts, the Netherlands) at their waist level, as shown in Figure 1a, for three consecutive days (72 h) and performed their activities of daily living in a usual manner. The device includes a tri-axial accelerometer (resolution of ±1 mg and range of ±6 g) and a tri-axial gyroscope (sensor resolution of ±0.0069°/s and range of ±100°/s). The data were transferred to the computer after the wearable device recorded the data into the in-built SD card of the device. Participants followed instructions to take off the sensor if there were any chances of getting wet or damaged. As per the researcher’s instructions, participants had to wear the sensor and orient/align at the low back (Figure 1a). The orientation of the sensor was such that when standing in a normal posture, the acceleration vector in the *y*-direction was vertically downwards. When standing in a normal position, the accelerations were a = [*a_x_*, *a_y_*, *a_z_*] = *g* [0, −1, 0] m/s^2^, where *g* = 9.81 m/s^2^. The sampling frequency of the sensor was set to 100 Hz. Along with acceleration, this IMU sensor also collected angular velocity ***ω*** = [*ω_x_*, *ω_y_*, *ω_z_*] in rad/s measured about each of the sensor-centric coordinate axes shown in Figure 1. Algorithms developed were entirely based on accelerometric data. We used Chapman University’s Keck Center for Science and Engineering for computation. The cluster contains over 590 Intel Xeon compute cores, 2.7 TB of RAM, and direct access to all-flash and disk-storage SAN arrays. Sixteen Nvidia TESLA V100 cards are available for workloads that benefit from GPU-accelerated processing. 

## 3. Results

**Figure 5:**
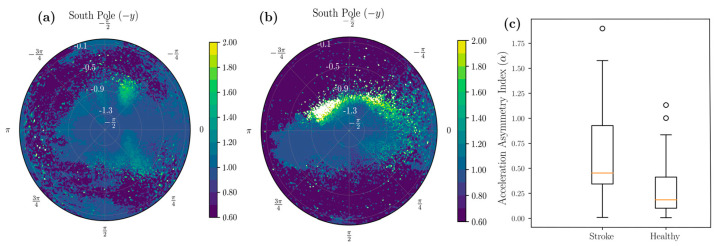
Visual comparison of symmetry in stroke versus healthy participants. (**a**) Distributions of mean accelerations in a healthy participant. (**b**) Distribution of means in a stroke participant. (**c**) Box plot representing acceleration asymmetry index (AAI) and stroke and healthy individuals.

The unit sphere projection technique allowed us to visualize various statistical aspects of the entire dataset in a single shot. Sensors were all worn in a similar configuration by patients, and direction-wise insights to patient behavior could be evaluated. Figure 3a,b shows the probability distribution of the data arising from a healthy and stroke participant across the northern and southern hemispheres of the UAS, respectively. Focusing on the southern hemisphere of the UAS, Figure 4a shows the distribution of the mean, and Figure 4b shows the standard deviation of various orientations of the IMU across the UAS. The visualization of statistics allows understanding which configurations are most common, have the highest amplitude, and depict the highest variability for the participant. We can differentiate two participants using these methods. Figure 5 shows the mean acceleration magnitude for healthy (Figure 5a) and post-stroke individuals (Figure 5b), and we can distinguish the asymmetry in their acceleration profile. Informed with this qualitative visual depiction of asymmetry, a quantitative measure of acceleration asymmetry index (α in Equation (5)) could be evaluated. Figure 5c shows this metric among post-stroke and healthy individuals. 

**Figure 6:**
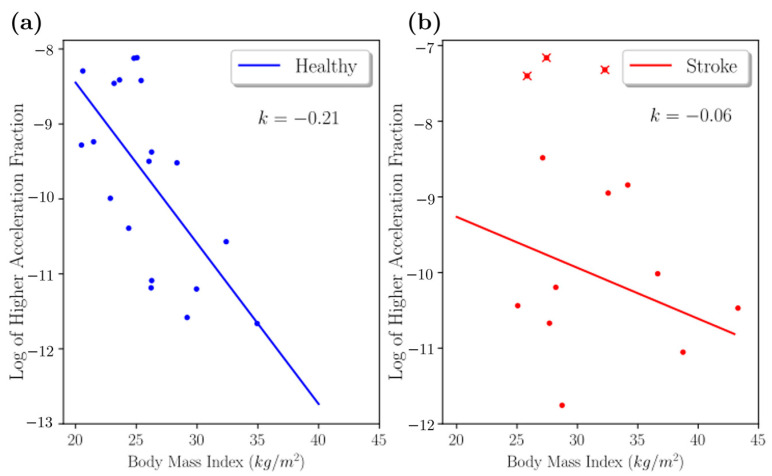
(**a**) Relationship between higher-acceleration fraction with healthy participant BMI. (**b**) Relationship between higher-acceleration fraction with stroke participant BMI. The figure shows three outliers as red x data points.

The ADL movements were analyzed during daytime (between 7 a.m. and 8 p.m., θ < −π/3). We found acceleration asymmetry index α was significantly higher in the stroke patients (mean = 0.61, SD = 0.44) than the healthy patients (mean = 0.24, SD = 0.20, t (27) = 2.59, *p* = 0.01 (Welch’s single-tailed independent two-sample t-test)) (Figure 5c). We found BMI was negatively correlated with high-acceleration movements (|a|>2g) (Figure 6). The negative slope was found as 0.21 units for healthy adults and 0.06 units for individuals with stroke. 

**Figure 7:**
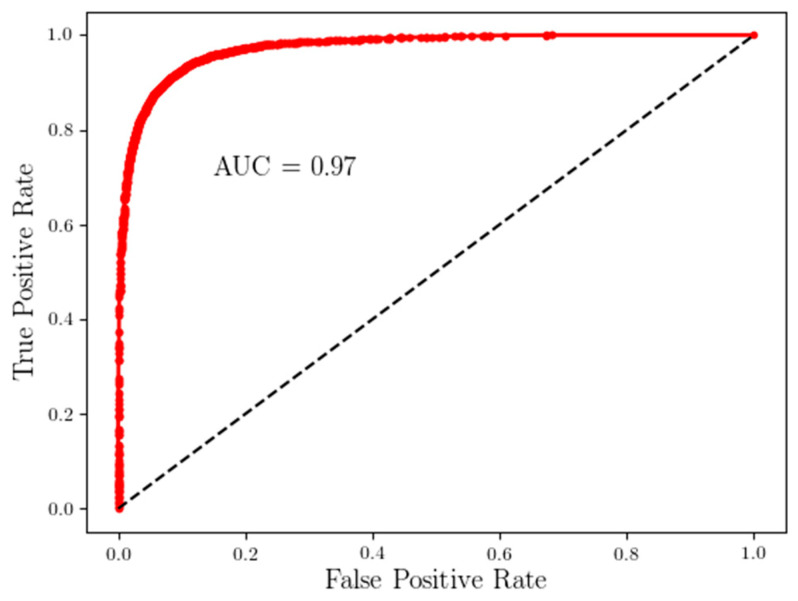
ROC curve of a deep LSTM model trained on information-rich time-series extracted from longitudinal multi-day data.

The ROC curve of a deep LSTM model trained on an information-rich time-series was extracted from the longitudinal data (Figure 7). The threshold of the final activation layer was adjusted to obtain the desired high sensitivity while keeping the false positives low, with the area under the ROC curve as 0.97.

**Figure 8:**
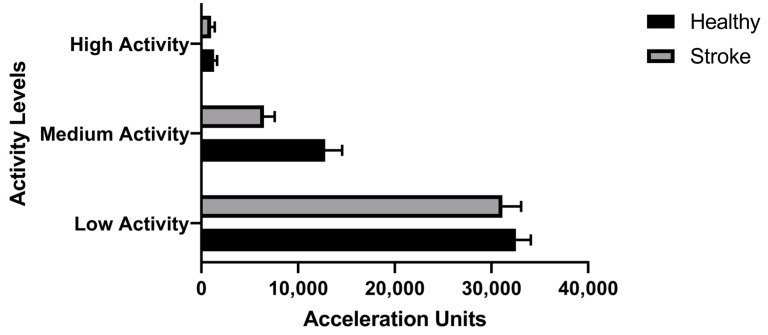
Total activity amplitudes (AAs) with low, medium, and high amplitudes among stroke and healthy adults.

**Figure 9:**
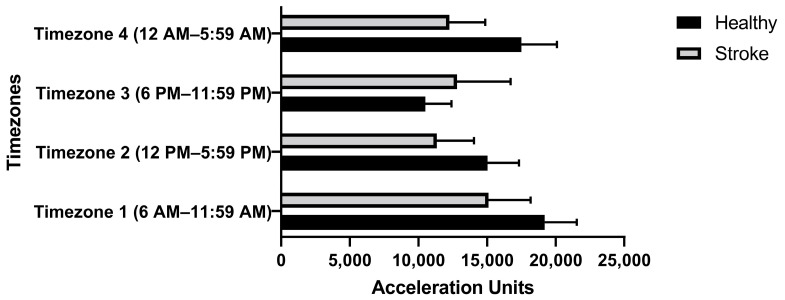
Time-zone-wise distribution of activity amplitude among stroke and healthy participants.

We found stroke participants produced fewer activity amplitudes than their healthy counterparts when compared amplitudes in three levels (low, medium, and high) (Figure 8). For most of the day, healthy participants were more active; however, stroke participants were more active during the evening (6 p.m.–11:59 p.m.) (Figure 9).

## 4. Discussion

This study introduces a new method to visualize longitudinal data that allows the extraction of information-rich time-series samples that can be used as input to other models. We demonstrate the usefulness of this technique by quantifying ADL movement differences among stroke and healthy adults. 

In this study, participants wear sensors at the low back and complete naturalistic activities in their home environments. Various functional and disability factors limit the participation of stroke survivors in essential and valued activities [26,27]. Previously, researchers have reported mobility decline, cognitive impairment, fatigue, difficulty in performing ADL, lack of communication and social interaction, and lack of self-efficacy to restrict movement among stroke survivors [28,29,30]. We found visually extracted time series as input was critical for the classification of stroke and healthy groups. The stroke affected daily activity movements, and recurrent neural networks such as LSTM trained on the extracted time series could classify stroke ADL movements with high sensitivity and specificity (AUC = 0.97) (Figure 7).

Wearable sensors such as accelerometers and gyroscopes produce massive volumes of health information. New technologies are needed to manage, analyze, and extract interpretable clinical data to improve patients’ quality of life. Modern advances in acute medical treatment of stroke have increased stroke survival rates during the last decade. However, some patients continue to decline in ADL performance after six months from stroke [31]. Thus, wearable-technology-assisted early identification of deteriorating patients will help occupational therapists provide adequate rehabilitation support and continuous monitoring of these patients to prevent further decline in ADL performance [32]. This study has innovatively utilized visual polar projections (Figure 2, Figure 3 and Figure 4) to select necessary informative IMU signals that could quantify stroke movements and help assess rehabilitation progress based on daily ADL performance. For instance, physical therapists treat stroke pathologies such as spasticity, range of motion, muscle strength, and pain. However, their treatments often result in increased physical capacity of stroke survivors and improved performance during ADL [33]. Still, no standard tool exists as of now to measure rehabilitation progress among stroke survivors. Cognitive deficits are also common after stroke and substantially affect stroke recovery and ADL performance [34,35,36]. These cognitive deficits negatively affect attention, memory, executive functioning, and motor control [37], affecting ties to perform ADL and participate in meaningful activities after stroke [34,35,36]. These deleterious effects of ADL on health outcomes and quality of life after stroke are well documented [29]. Previous studies suggest younger age, decreased stroke severity, and better motor and functional abilities at stroke onset are correlated with ADL recovery beyond three months after stroke [38,39].

Asymmetry among Stroke Survivors: Asymmetry after stroke is a salient index of movement dysfunction and has negative functional consequences. We found that the stroke participants exhibited higher asymmetry in their accelerations and movements, e.g., acceleration asymmetry index *α* between the two groups, with the stroke group having significantly more asymmetry (*p* = 0.01) (Figure 5). These asymmetry measures from wearable sensors have predictive value [40,41] of assessing global movement and stroke-related deficits.

Influence of BMI: Stroke leads to paralysis [42,43], resulting in a reduction in muscle mass and an increase in fat mass [44,45]. These physiological muscle changes lead to deficits in the body function of stroke survivors [46]. This loss of body function will ultimately affect the accelerations developed during the movement. Sensors detected ADL-related tasks that patients performed daily, such as eating, dressing, toileting, and ambulation. We have earlier reported that obesity affects movement transitions [23]. Similar trends were observed among healthy and stroke individuals (Figure 6). Our results revealed a negative correlation of BMI with high amplitude accelerations. These trends were more pronounced in the healthy group in contrast to the stroke group. This may be due to heterogeneity among stroke survivors and different levels of stroke severity (Table 3). 

Sedentary Behavior: We found stroke activity amplitudes were lower in all three activity bands (low, medium, and high) (Figure 8). These results revealed an overall decrease in activities (all types of activities: low, medium) or adoption of sedentary lifestyles in stroke survivors. We found healthy participants to be active throughout the day, but stroke participants were found to be slightly more involved in the evening (6 a.m. to 11:59 p.m.) (Figure 9). This could be partly attributed to participation in stroke rehabilitation evening community programs. Understanding trends of movement accelerations among individuals with stroke could help develop rehabilitation regimens matching activity levels of these individuals to promote physical activities.

In fact, the two features φ and θ can be converted into important visual information. These features can discretize the UAS into bins on which various statistics can be computed. When we visualize polar coordinates with the azimuthal angle along the angular coordinate and the polar angle along the radial coordinate (Figure 3), we can connect the physical orientation of the acceleration vector with the sensor orientation. This visualization provides insights into the distribution of the movement data. For example, high probability areas or activities can be represented on the UAS in Figure 3. These are orientations where the subjects spend a lot of time. However, when we combine them with the mean and standard deviation statistics, we find that the high probability zones might not contain the high-acceleration movements. The spatial distribution of the high standard deviation and high mean regions is of interest to us, as it contains movements that correspond to repeated information-rich samples. The projection on the sphere and the knowledge of how the subjects wore the sensor gives us a visual understanding of asymmetry in the movement characteristics by looking at the entire dataset in one single view. In addition to the qualitative look at the data, we developed data-filtering criteria based on the mean and variability distribution on the UAS to provide training samples to LSTM, obtain asymmetry information, and gain further insights into the correlation of BMI with activity performance.

We should interpret the results with the limitations of this study. Although three days of sensor data were voluminous, the sample size was small for machine learning (14 healthy and 14 stroke individuals). Thus, the LSTM model may be overfitting to these idiosyncrasies of movements of these individuals. With the enrollment of more subjects, we can put the reliability of the time-series extraction methods through a more robust analysis by training it on one population of subjects and testing models on a completely new population dataset. Similarly, the asymmetry and correlation of activity with BMI results can be more robust with more subjects. The development of these images requires computational resources. When we have to develop the training set for 100s of patients, this task might require up to two to three days on a cluster to develop the images. The strength of this study is developing a new method to analyze passive data collected from participants’ naturalistic environments. This maximizes the generalizability of these algorithms in detecting activities from real-world settings.

Traditionally a spectrogram of the entire dataset is considered the first choice to run CNNs to classify the images [47]. Although spectrograms provide important information about frequencies involved during movement, they fail to quantify movement. We envision using the UAS (Figure 2, Figure 3 and Figure 4) in regular 2D matrix form could potentially open new avenues in ADL identification and quantification methods. Statistics computed on the UAS can serve as an input channel to CNN. Thus, encompassing multiple statistics (Figure 2) can either be substituted or be used in conjunction with the spectrogram approach to enrich the identification and classification of ADL tasks from longitudinal time-series data.

## 5. Conclusions

Understanding asymmetry of movements during ADL performance in natural environments is a very consequential goal for stroke rehabilitation. Currently, there is a paucity of knowledge on how passively collected wearable sensor data could provide important health-related information. Deploying visual presentation of passively collected wearable sensor data in polar coordinates and utilizing this information to filter information-rich data samples to provide as input to machine learning models is highly promising for automated objective quantification of ADL performance. In-home assessment of activities for stroke survivors will help clinicians to design interventions personalized to their everyday life.

The use of wearable sensors, visual algorithms for time-series extraction, and multi-day motion tracking will allow patient assessment more realistically in their home environments. The wearable sensors would enable patients to perform ADL unobtrusively and independently. With new methods of processing multiple-day motion tracking data, we are entering into a new era of remote monitoring of functional performance in stroke survivors. Remote monitoring of functional activity assessment will provide insights to clinicians of the efficacy of stroke rehabilitation programs.

## Figures and Tables

**Table 1:** Mean, standard deviation, and effect size of features.

Features	Healthy (μ, σ)	Stroke (μ, σ)	Effect Size (Cohen’s d)
$a_{x}$	−0.01, 0.14	0.01, 0.15	0.16
$a_{y}$	−0.93, 0.14	−0.91, 0.14	0.16
$a_{z}$	0.05, 0.33	0.14, 0.35	0.28
ω_x_	−0.06, 13.82	−0.29, 15.15	−0.02
ω_y_	−0.60, 28.18	0.34, 19.49	0.04
ω_z_	−0.20, 12.09	−0.13, 9.98	0.01
|a|	1.00, 0.11	1.00, 0.09	0.01
$a_{xs}$	−0.01, 0.13	0.01, 0.15	0.17
$a_{ys}$	−0.93, 0.09	−0.91, 0.11	0.24
$a_{zs}$	0.05, 0.33	0.14, 0.35	0.28
ϕ	−0.30, 2.03	0.09, 2.19	0.19
θ	−1.24, 0.20	−1.19, 0.22	0.26

**Table 2:** The input, output, and hidden layers of the recurrent neural network.

Layer	Output Shape	Parameters
LSTM	(None,300,200)	170,400
Batch Normalization	(None,300,200)	800
LSTM	(None,50)	50,200
Dropout	(None,50)	0
Dense (ReLU)	(None,50)	2550
Dropout	(None,50)	0
Dense (ReLU)	(None,15)	765
Dense (Sigmoid)	(None,1)	16

**Table 3:** Anthropometric data of individuals with stroke and healthy adults.

	Stroke	Control
Age (years)	69 ± 8.4	74 ± 8.7
BMI (kg/m^2^)	30.8 ± 5.6	26.1 ± 3.0
Gender	Six females and eight males	Eight females and six males

**Table 4:** Fugl–Meyer scores of fourteen healthy and fourteen stroke participants.

Fugl–Meyer Scores	Stroke	Healthy
Lower Extremity Score	18.5 ± 3.3	28 ± 0
Coordination Speed	4.1 ± 1.0	6 ± 0
Motor Function	22.7 ± 3.7	34 ± 0
Sensation Score	9.2 ± 3.4	12 ± 0
Passive Joint Motion	15.7 ± 2	20 ± 0
Joint Pain	19.7 ± 0.5	20 ± 0

## Data Availability

The raw data supporting the conclusions of this article are available at https://doi.org/10.36837/chapman.000334, accessed on 1 November 2021.
